# Hydrogen exerts neuroprotection by activation of the miR‐21/PI3K/AKT/GSK‐3β pathway in an in vitro model of traumatic brain injury

**DOI:** 10.1111/jcmm.15051

**Published:** 2020-02-28

**Authors:** Lu Wang, Zhenyu Yin, Feng Wang, Zhaoli Han, Yifeng Wang, Shan Huang, Tianpeng Hu, Mengtian Guo, Ping Lei

**Affiliations:** ^1^ Department of Geriatrics Tianjin Medical University General Hospital Tianjin Geriatrics Institute Tianjin China; ^2^ Department of Intensive Care Unit Tianjin Medical University General Hospital Tianjin China

**Keywords:** apoptosis, GSK‐3β, hydrogen, miR‐21, nerve regeneration, traumatic brain injury

## Abstract

Few studies have explored the effect of hydrogen on neuronal apoptosis or impaired nerve regeneration after traumatic brain injury, and the mechanisms involved in these processes are unclear. In this study, we explored neuroprotection of hydrogen‐rich medium through activation of the miR‐21/PI3K/AKT/GSK‐3β pathway in an in vitro model of traumatic brain injury. Such model adopted PC12 cells with manual scratching. Then, injured cells were cultured in hydrogen‐rich medium for 48 hours. Expression of miR‐21, p‐PI3K, p‐Akt, p‐GSK‐3β, Bax and Bcl‐2 was measured using RT‐qPCR, Western blot analysis and immunofluorescence staining. Rate of apoptosis was determined using TUNEL staining. Neuronal regeneration was assessed using immunofluorescence staining. The results showed that hydrogen‐rich medium improved neurite regeneration and inhibited apoptosis in the injured cells. Scratch injury was accompanied by up‐regulation of miR‐21, p‐PI3K, p‐Akt and p‐GSK‐3β. A miR‐21 antagomir inhibited the expression of these four molecules, while a PI3K blocker only affected the three proteins and not miR‐21. Both the miR‐21 antagomir and PI3K blocker reversed the protective effect of hydrogen. In conclusion, hydrogen exerted a neuroprotective effect against neuronal apoptosis and impaired nerve regeneration through activation of miR‐21/PI3K/AKT/GSK‐3β signalling in this in vitro model of traumatic brain injury.

## 1 INTRODUCTION

Traumatic brain injury (TBI) is an extremely common condition, affecting people of all ages and genders.^1^ In the United States, about 1.7‐2 million people suffer from TBI each year.^2^ The incidence of TBI in China is lower than that in the United States, but the annual number of cases in China has nearly doubled in recent years.^3^ Furthermore, TBI is the leading form of brain injury in children, and the main cause of disability and death in young and middle‐aged people in most countries.^1^ Because of this, costs of TBI to families and to society are relatively high all around the world.

It is well known that TBI causes progressive damage in brain tissue, such as blood‐brain barrier leakage, brain oedema and intracranial hypertension,^4^ which may partly be induced or promoted by oxidative stress, neuronal apoptosis and many other cellular/molecular dysfunctions.^5^ These pathological abnormalities at tissue, cellular or molecular levels eventually affect neurons, resulting in quantity reduction, structural change and subsequent neurological dysfunction.^5^ Therefore, it is of great significance to alleviate the cellular and molecular abnormalities after TBI.

The medical value of hydrogen in brain injury is gradually being recognized. Studies by ourselves and others have found that hydrogen gas or hydrogen‐rich water improves blood‐brain barrier leakage, relieves brain oedema and inhibits oxidative stress in rat models of TBI.^6^, ^7^, ^8^ Some studies confirmed that hydrogen suppresses neuronal apoptosis in animal models of other brain injuries (ie septic, anaesthetic and ischaemic brain injuries), but not in models of TBI.^9^, ^10^, ^11^ Furthermore, few studies have explored improvement in nerve regeneration after hydrogen treatment in any type of brain injury. Therefore, the multiple protective effects of hydrogen on TBI should be further investigated.

Our previous study suggested that increased expression of microRNA‐21 (miR‐21) may play a crucial role in the neuroprotective effects of hydrogen treatment and that down‐regulation of miR‐21 may reverse such effects after TBI.^6^ However, more detailed molecular mechanisms remained unclear. Many studies indicate that miR‐21 can activate the PI3K/AKT signalling pathway in models of Alzheimer's disease and several malignant tumours.^12^, ^13^, ^14^ Other studies found that activation of the AKT/GSK‐3β signalling pathway attenuated oxidative stress and apoptosis in models of Alzheimer's disease, Parkinson's disease and intracerebral haemorrhage.^15^, ^16^, ^17^ More importantly, oxidative stress and apoptosis were common molecular abnormalities induced by TBI. Therefore, we suggested that miR‐21/PI3K/AKT/GSK‐3β signalling is involved in the protective effects of hydrogen on TBI.

In this study, we adopted an in vitro model of TBI to reveal the protective effects of hydrogen‐rich medium on neuronal apoptosis, oxidative stress and nerve regeneration ability, and to explore the molecular mechanisms involved.

## 2 MATERIALS AND METHODS

The study was approved by the ethics committees of Tianjin Medical University General Hospital (Tianjin, China).

### 2.1 Cell line and culture

PC12 cells were purchased from Shanghai Sixin Biotechnology and cultured according to the routine procedures. They were maintained in high sugar Dulbecco's modified Eagle's medium (DMEM) (Sigma‐Aldrich) with 10% foetal bovine serum and 0.5% penicillin/streptomycin at constant temperature (37°C) in a 5% carbon dioxide incubator (Thermo Fisher Scientific).

PC12 cells were cultured to monolayers on 6‐well plates for further experiments. In the first series of experiments, the cells were randomly divided into control group, control + hydrogen intervention group (Con + H_2_ group), control + miR‐21 inhibition group (Con + M group) and control + PI3K inhibition group (Con + P group). Cell viability, neuronal injury, apoptosis rate and oxidative stress indicators were compared among these groups to evaluate the effects of hydrogen and the miR‐21 and PI3K inhibitors on normal PC12 cells. In the second series of experiments, the cells were randomly divided into control group, TBI group, hydrogen intervention group (TBI + H_2_ group), miR‐21 inhibition group (miR21_I group) and PI3K inhibition group (PI3K_I group). Neuroprotection by hydrogen and its molecular mechanism in TBI was explored. Each group included 10 wells of PC12 cells.

### 2.2 Transfection and inhibition

MiR‐21 antagomir transfection was performed 6 hours before TBI modelling. The miR‐21 antagomir, obtained from GenePharma, was transfected into Con + M group and miR21_I group PC12 cells using Lipofectamine (Lip) 3000 reagent according to the manufacturer's instructions (Invitrogen). Briefly, PC12 cells were cultured for 24 hours to reach about 50% confluence and were then transfected with 50 µmol/L miR‐21 antagomir. Stably transfected clones were selected using G418 (Sigma‐Aldrich, Merck KGaA). PC12 cells in other groups were not transfected with miR‐21 antagomir.

PC12 cells in Con + P and PI3K_I groups were treated with 50 μmol/L LY294002 (MedChem Express), a PI3K inhibitor, 30 minutes before TBI modelling. PC12 cells in other groups were not treated with this reagent.

### 2.3 Modelling

A widely accepted method was adopted to establish an in vitro model of TBI in the TBI, TBI + H_2_, miR21_I and PI3K_I groups.^18^, ^19^, ^20^ Briefly, monolayers of PC12 cells in 6‐well plates were manually scratched with 10‐μl micropipette tips, and the space between each scratch was about 4 mm. Cells in the control group did not undergo this intervention.

### 2.4 Hydrogen‐rich medium

Hydrogen‐rich DMEM was prepared using a widely adopted method with some modifications.^21^ A pure and pressurized hydrogen environment (purity greater than 99.9999%, pressure at 0.4 MPa) was created by a GCH‐500 hydrogen generator (Tongpu Analytical Instrument Technology). DMEM (Gibco) was exposed to this environment for 4 hours until hydrogen had dissolved to saturation. The concentration of hydrogen in the medium was measured using a HY‐ALERTA™ 500 hydrogen electrode (H2scan). If it reached or exceeded 6 mmol/L, the medium was temporarily stored at 4°C in a sealed aluminium bag after filtration and sterilization. All hydrogen‐rich media were fleshly prepared and used in experiments as soon as possible.

After scratching, PC12 cells in the TBI + H_2_, miR21_I and PI3K_I groups were cultured in hydrogen‐rich medium for 48 hours. The cells in the Con + H_2_ group were also cultured in such medium for 48 hours.

### 2.5 Reverse transcription‐quantitative PCR

The level of miR‐21 in control cells or injured cells 48 hours after scratching was measured using reverse transcription‐quantitative PCR. Total RNA was extracted using TRIzol reagent (Invitrogen) and transcribed into cDNA using a Hairpin‐it™ miRNA RT‐PCR Quantitation Kit or a U6 snRNA Real‐time RT‐PCR Normalization Kit (GenePharma) according to the manufacturer's instructions. Forward and reverse primer sequences of miR‐21 were 5′‐ACGTTGTGTAGCTTATCAGACTG‐3′ and 5′‐AATGGTTGTTCTCCACACTCTC‐3′, respectively. U6 snRNA was adopted as an endogenous control, and its forward and reverse primer sequences were as follows: 5′‐ATTGGAACGATACAGAGAAGATT‐3′ and 5′‐GGAACGCTTCACGAATTTG‐3′, respectively. Forty cycles of PCR were performed, and each cycle included three steps: denaturation (95°C for 3 minutes), annealing (62°C for 30 seconds) and elongation (72°C for 30 seconds). Relative levels of miR‐21 are expressed using 2^−△△Ct^ values.

### 2.6 Western blot analysis

Levels of p‐PI3K, p‐Akt, p‐GSK‐3β, Bax and Bcl‐2 proteins were measured by Western blot analysis in control and injured cells 48 hours after scratching. Total protein concentrations were determined using a Pierce BCA Protein Assay Kit (Solarbio) according to the manufacturer's instructions. Equal amounts of protein (100 μg) were separated using 12% sodium dodecyl sulphate‐polyacrylamide gel electrophoresis (SDS‐PAGE), transferred onto a polyvinylidene fluoride (PVDF) membrane (Millipore), blocked using 5% skimmed milk in Tris‐buffered saline for 2 hours and then incubated overnight at 4°C with primary antibodies. Antibodies used were as follows: anti‐PI3K (1:1000 dilution), anti‐p‐PI3K (1:1000 dilution), anti‐Akt (1:1000 dilution), anti‐p‐Akt (1:1000 dilution), anti‐GSK‐3β (1:1000 dilution), anti‐p‐GSK‐3β (1:1000 dilution), anti‐Bax (1:1000 dilution), anti‐Bcl‐2 (1:1000 dilution) and anti‐GAPDH (1:1000 dilution). All primary antibodies were purchased from Cell Signaling Technology. Then, the membranes were washed three times using Tris‐buffered saline and incubated with horseradish peroxidase (HRP)–conjugated goat anti‐rabbit secondary antibodies (1:10,000 dilution, Cell Signaling Technology) for 1 hour at room temperature. Chemiluminescent HRP substrate was used to visualize the protein bands (Immobilon Western, Millipore). Relative intensities of proteins vs GAPDH bands were measured using ImageJ software.

### 2.7 MTT assay

Neuronal viability and growth were evaluated using a commercial 3‐(4, 5‐dimethylthiazol‐2‐yl)‐2, 4,‐diphenyltetrazolium bromide (MTT) proliferation assay kit (Invitrogen) in control cells and injured cells 48 hours after scratching. Briefly, PC12 cells were seeded in 96‐well plates and incubated with substrate for 2 hours. Then, isopropyl alcohol was added for a further 2‐hour incubation. Cell lysates were collected, and their optical density (OD) at 570 nm (test wavelength) and 620 nm (reference wavelength) was measured using a microplate photometer (Thermo Fisher Scientific). Percentage neuronal viability was calculated using the formula: OD at 570 nm/OD at 620 nm × 100%.

### 2.8 LDH assay

Lactate dehydrogenase (LDH) can be released into culture medium after cell injury or death; therefore, neuronal injury in control cells and injured cells 48 hours after scratching was evaluated by measuring LDH concentration in culture medium using a commercial LDH detection kit (Biyuntian Biotechnological Technology). The procedure was conducted according to the manufacturer's recommendations. OD at 490 nm was measured using a microplate photometer (Thermo Fisher Scientific). Results are expressed as the percentage of control values.

### 2.9 Double immunofluorescence and TUNEL staining

Double immunofluorescence staining for GSK‐3β was performed in control cells and injured cells 48 hours after scratching to determine GSK‐3β expression. Treated cells were fixed using paraformaldehyde solution (4%) and permeabilized using Triton X‐100 solution (0.1%). Then, the cells were incubated with an anti‐GSK‐3β primary antibody at 4°C overnight (1:300 dilution, Sigma‐Aldrich). After that, the cells were incubated with a Alexa Fluor 488‐conjugated goat antimouse secondary antibody (Thermo Fisher Scientific) at room temperature for 1 hour. Nuclei were stained using 4, 6‐diamidino‐2‐phenylindole (DAPI). Mean fluorescence intensity was measured using ImageJ software.

Double immunofluorescence staining for βIII‐tubulin and TUNEL staining was also performed on control cells and injured cells to detect neuronal regeneration and neuronal apoptosis. Briefly, βIII‐tubulin immunostaining was conducted with an anti‐βIII‐tubulin primary antibody (1:300 dilution, Sigma‐Aldrich) and then incubated with a TRITC‐(tetra‐methyl‐5, 6‐isothiocyanate)‐labelled phalloidin secondary antibody (Thermo Fisher Scientific). Subsequently, control and injured cells were reacted for 2 hours with the TUNEL reacting mixture from the TUNEL Kit (Roche). Finally, DAPI was used to stain nuclei. Numbers of βIII‐tubulin‐positive, TUNEL‐positive and total neurons were manually counted at 200× magnification.

### 2.10 Measurement of SOD, CAT and MDA

Oxidative stress levels were assessed by determining superoxide dismutase (SOD), catalase (CAT) and malondialdehyde (MDA) levels in control cells and injured cells 48 hours after scratching. Cells in all groups were centrifuged at 100 *g* for 5 minutes, and their supernatants were obtained. Commercial test kits (Cayman Chemical) were used to measure the levels of SOD, CAT and MDA according to the manufacturer's instructions. Optical densities were determined at 570 nm using a DU 640B spectrophotometer (Beckman).

### 2.11 Statistical analysis

Continuous variables are expressed in the form of the mean ± standard deviation (SD). Difference between two continuous variables was measured using the independent sample *t* test. Difference among more than two continuous variables was determined using one‐way ANOVA with the LSD *t* test. *P* value < .05 indicates statistical significance. All statistical analyses were conducted using SPSS 18.0 software.

## 3 RESULTS

### 3.1 Normal cells were not affected by hydrogen or by the miR‐21 and PI3K inhibitors

**Figure 1:**
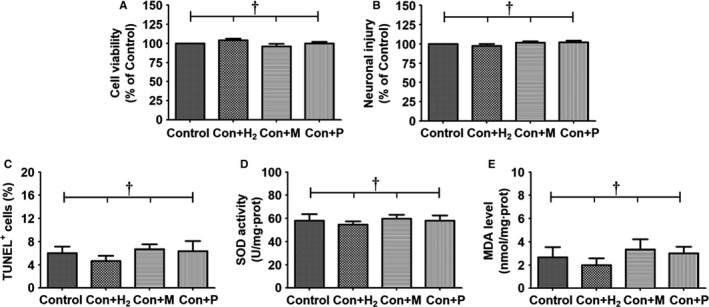
Effects of hydrogen gas and two reagents on normal PC12 cells. Prepared PC12 cells that were cultured on 6‐well plates were randomly divided into control group, control + hydrogen intervention group (Con + H_2_ group), control + miR‐21 inhibition group (Con + M group) and control + PI3K inhibition group (Con + P group). Each group included 10 wells of PC12 cells. The cells in the Con + H_2_ group were cultured in hydrogen‐rich DMEM for 48 h. MiR‐21 antagomir was transfected into PC12 cells in the Con + M group. The cells in the Con + P group were treated with PI3K blocker LY294002 for 30 min. After these interventions, cell viability was evaluated using a commercial MTT proliferation assay kit. Neuronal injury was evaluated by measurement of lactate dehydrogenase (LDH) concentration using a commercial LDH detection kit. Percentage of TUNEL^+^ cells was obtained using a commercial TUNEL kit. Levels of superoxide dismutase (SOD) and malondialdehyde (MDA) in PC12 cells were measured using their commercial test kits. The results were expressed as mean ± standard deviation. Differences in these markers among the four groups were compared using one‐way ANOVA with LSD *t* test. ‘†’ indicates *P* > .05

As shown in Figure 1, there was no significant difference in cell viability among control, Con + H_2_, Con + M and Con + P groups (*P* > .05). Furthermore, neuronal injury, apoptosis rate, levels of SOD and MDA were equivalent among these four groups (*P* > .05, *P* > .05, *P* > .05 and *P* > .05, respectively). These findings confirmed that hydrogen and the miR‐21 and PI3K inhibitors (miR‐21 antagomir and LY294002) did not affect cell viability, neuronal injury, apoptosis rate or oxidative stress in normal PC12 cells.

### 3.2 Hydrogen intervention elevates the expression of miR‐21, p‐PI3K, p‐Akt and p‐GSK‐3β in injured cells

**Figure 2:**
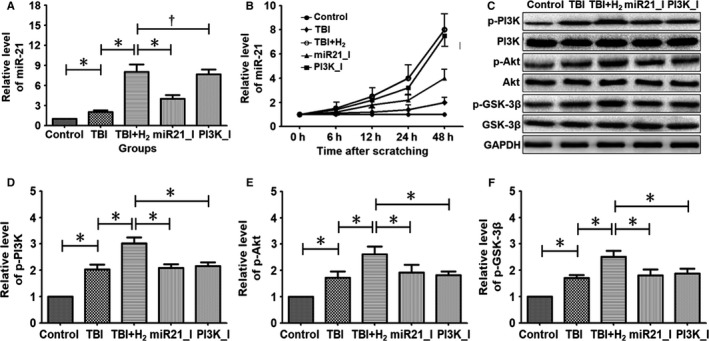
Expressions of miR‐21 and several proteins in PC12 cells. Prepared PC12 cells were randomly divided into control group, TBI group, hydrogen intervention group (TBI + H2 group), miR‐21 inhibition group (miR21_I group) and PI3K inhibition group (PI3K_I group). Each group included 10 wells of PC12 cells. Before scratching, miR‐21 antagomir was transfected into PC12 cells in the miR21_I group, and PC12 cells in the PI3K_I group were treated with PI3K blocker LY294002. After scratching, the injured cells in the TBI + H2 group, miR21_I group and PI3K_I group were cultured in hydrogen‐rich DMEM for 48 h. Expression levels of miR‐21, p‐PI3K, p‐Akt and p‐GSK‐3β were measured using reverse transcription–quantitative PCR, Western blot analysis. Quantitative results were expressed as mean ± standard deviation. Differences in these markers among the five groups were compared using one‐way ANOVA with LSD *t* test. ‘*’ indicates *P *< .05 and ‘†’ indicates *P* > .05

Figure 2 shows differences in the levels of miR‐21, p‐PI3K, p‐Akt and p‐GSK‐3β among the control, TBI and TBI + H_2_ groups. In Figure 2A, the level of miR‐21 at 48 hours after scratching was higher in the TBI group than in the control group (*P* < .05). After hydrogen intervention, the level of miR‐21 was further elevated in the TBI + H_2_ group compared with the TBI group (*P* < .05). Figure 2B shows the dynamic changes in miR‐21 levels from 0 hour to 48 hours after scratching in these groups. The levels of p‐PI3K, p‐Akt and p‐GSK‐3β gradually increased in the control, TBI and TBI + H_2_ groups (Figure 2C‐F; control vs TBI: *P* < .05, *P* < .05 and *P* < .05, respectively; TBI vs TBI + H_2_: *P* < .05, *P* < .05 and *P* < .05, respectively).

**Figure 3:**
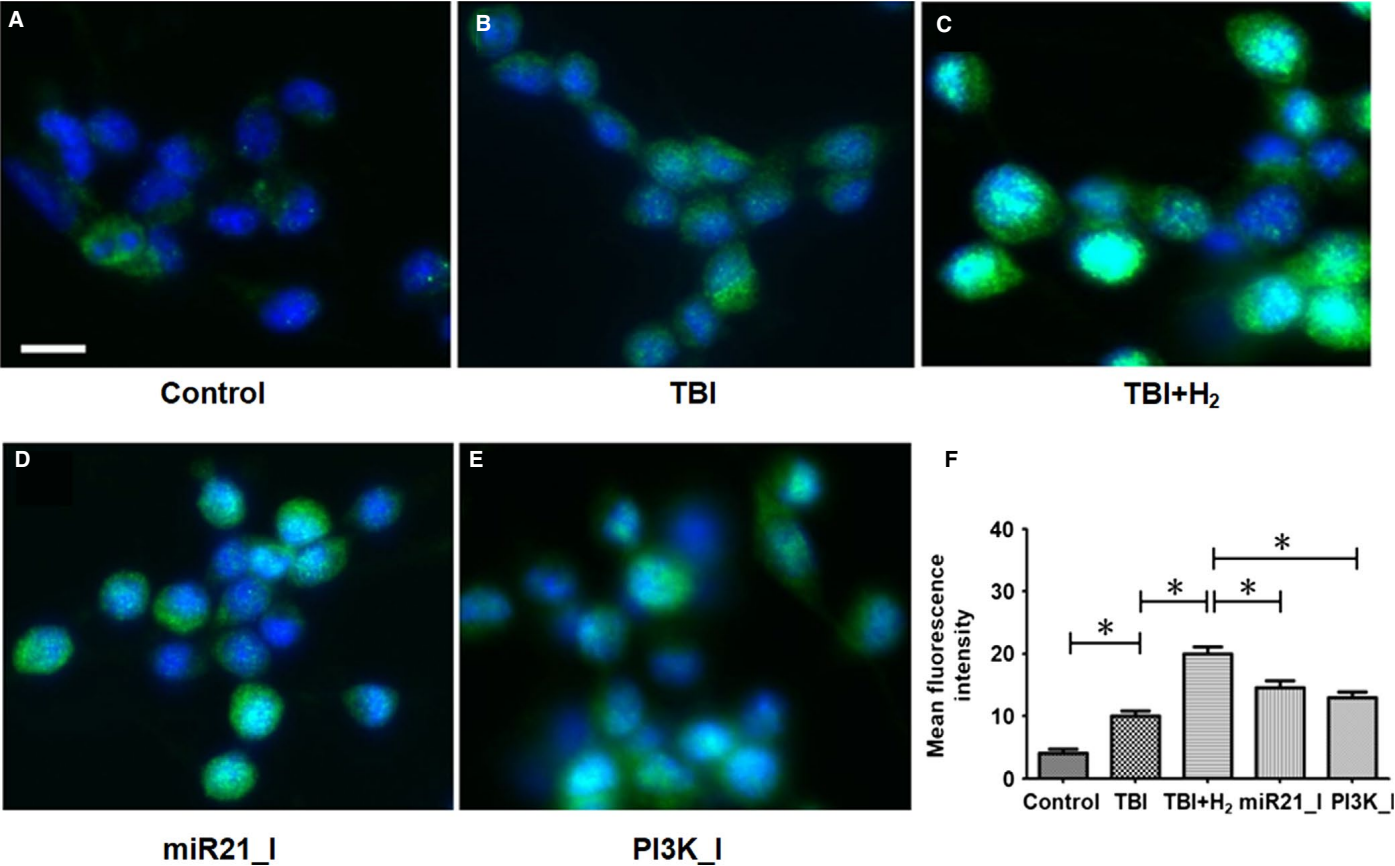
Expressions of p‐GSK‐3β in PC12 cells. Expression levels of p‐GSK‐3β were measured using double immunofluorescence staining. Mean fluorescence intensity was measured using ImageJ software. Quantitative results were expressed as mean ± standard deviation. Differences in these markers among the five groups were compared using one‐way ANOVA with LSD *t* test. ‘*’ indicates *P* < .05. Scale bar in Figure 3 is 5 μm

The mean fluorescence intensity of p‐GSK‐3β was sequentially elevated in control, TBI and TBI + H_2_ groups (Figure 3A‐C, *P* < .05 and *P* < .05, respectively). When the expression level of p‐GSK‐3β was relatively low, it was mainly distributed in the cytoplasm. With increasing expression, p‐GSK‐3β appeared in the nucleus.

These findings confirmed the up‐regulation of miR‐21, p‐PI3K, p‐Akt and p‐GSK‐3β in the TBI model and that hydrogen treatment after TBI can further up‐regulate the expression of these nucleic acids and proteins.

### 3.3 Existence of the miR‐21/PI3K/Akt/GSK‐3β signalling pathway in PC12 cells

The effects of the miR‐21 antagomir and PI3K blocker, LY294002, on the levels of miR‐21, p‐PI3K, p‐Akt and p‐GSK‐3β in PC12 cells were explored (Figure 2). Compared with the TBI + H_2_ group, the level of miR‐21 was decreased in the miR21_I group (*P* < .05), but there was no difference in the level of miR‐21 between the TBI + H_2_ and PI3K_I groups (Figure 2A, *P* > .05). Compared with the TBI + H_2_ group, the levels of p‐PI3K, p‐Akt and p‐GSK‐3β were significantly lower in the miR21_I and PI3K_I groups (Figure 2C‐F; miR21_I group: *P* < .05, *P* < .05 and *P* < .05, respectively; PI3K_I group: *P* < .05, *P* < .05 and *P* < .05, respectively). Compared with the TBI + H_2_ group, the mean fluorescence intensity of p‐GSK‐3β was significantly decreased in the miR21_I and PI3K_I groups (Figure 3C‐E, *P* < .05 and *P* < .05, respectively). These findings revealed that miR‐21 and GSK‐3β may be relative upstream and downstream factors and that PI3K/Akt is located in the middle of the pathway. Therefore, the signalling pathway miR‐21/PI3K/Akt/GSK‐3β indeed existed in PC12 cells.

### 3.4 Hydrogen improved cell viability and neuronal injury in injured cells partly through activation of the miR‐21/PI3K/Akt/GSK‐3β signalling pathway

**Figure 4:**
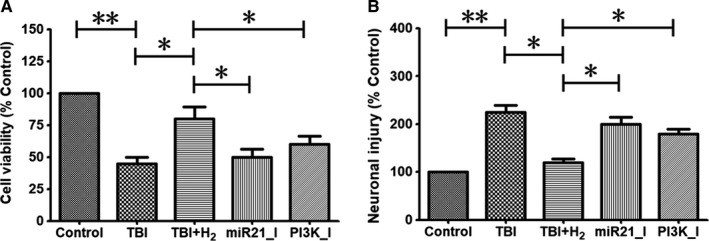
Cell viability and neuronal injury in PC12 cells. Cell viability was evaluated using a commercial MTT proliferation assay kit in control cells or injured cells 48 h after scratching. Neuronal injury was evaluated by measurement of lactate dehydrogenase (LDH) concentration using a commercial LDH detection kit at the same time point. The results were expressed as mean ± standard deviation. Differences in these markers among the five groups were compared using one‐way ANOVA with LSD *t* test. ‘*’ indicates *P* < .05, and ‘**’ indicates *P* < .01

Cell viability and neuronal injury were determined using MTT assays and LDH release rate, respectively. Cell viability was decreased in the TBI group compared with that in the control group (*P* < .01), and this decrease was reversed in the TBI + H_2_ group compared with the TBI group (Figure 4A, *P* < .05). In the miR21_I and PI3K_I groups, cell viability was less than that in the TBI + H_2_ group (Figure 4A, *P* < .05 and *P* < .05, respectively).

As illustrated in Figure 4B, LDH release rate was elevated in the TBI group compared with the control group (*P* < .01), and this decrease was reversed in the TBI + H_2_ group compared with the TBI group (*P* < .05). In the miR21_I and PI3K_I groups, LDH release rate was increased compared with that in the TBI + H_2_ group (*P* < .05 and *P* < .05, respectively). These findings indicated that hydrogen improved cell viability and repaired neuronal injury partly through activation of the miR‐21/PI3K/AKT/GSK‐3β pathway.

### 3.5 Hydrogen promoted regeneration of injured cells partly through activation of the miR‐21/PI3K/AKT/GSK‐3β signalling pathway

**Figure 5:**
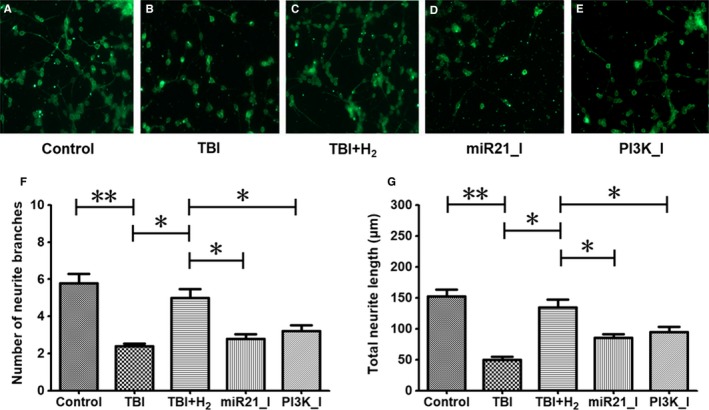
Neuronal injury regeneration in PC12 cells. Immunofluorescence staining for βIII‐tubulin was adopted in control cells or injured cells 48 h after scratch to detect neuronal regeneration. Number of neurite branches and total neurite length indicated neuronal injury regeneration ability. The results were expressed as mean ± standard deviation. Differences in these markers among the five groups were compared using one‐way ANOVA with LSD *t* test. ‘*’ indicates *P* < .05, and ‘**’ indicates *P* < .01

Figure 5A‐E shows immunofluorescence of PC12 cells in the control, TBI, TBI + H_2_, miR21_I and PI3K_I groups. The number of neurite branches was decreased, and total neurite length was shorter in the TBI group than in the control group (Figure 5F,G, *P* < .01 and *P* < .01, respectively). After hydrogen treatment, the number of neurite branches was increased and total neurite length was longer in the TBI + H_2_ group than in the TBI group (*P* < .05 and *P* < .05, respectively). However, the number of neurite branches and total neurite length were decreased in the miR21_I and PI3K_I groups compared with the TBI + H_2_ group (miR21_I vs TBI + H_2_: *P* < .05 and *P* < .05, respectively; PI3K_I vs TBI + H_2_: *P* < .05 and *P* < .05, respectively). These findings showed that hydrogen promoted regeneration after neuronal injury partly through activation of miR‐21/PI3K/AKT/GSK‐3β signalling in injured cells.

### 3.6 Hydrogen inhibits apoptosis in injured cells partly through activation of the miR‐21/PI3K/AKT/GSK‐3β signalling pathway

**Figure 6:**
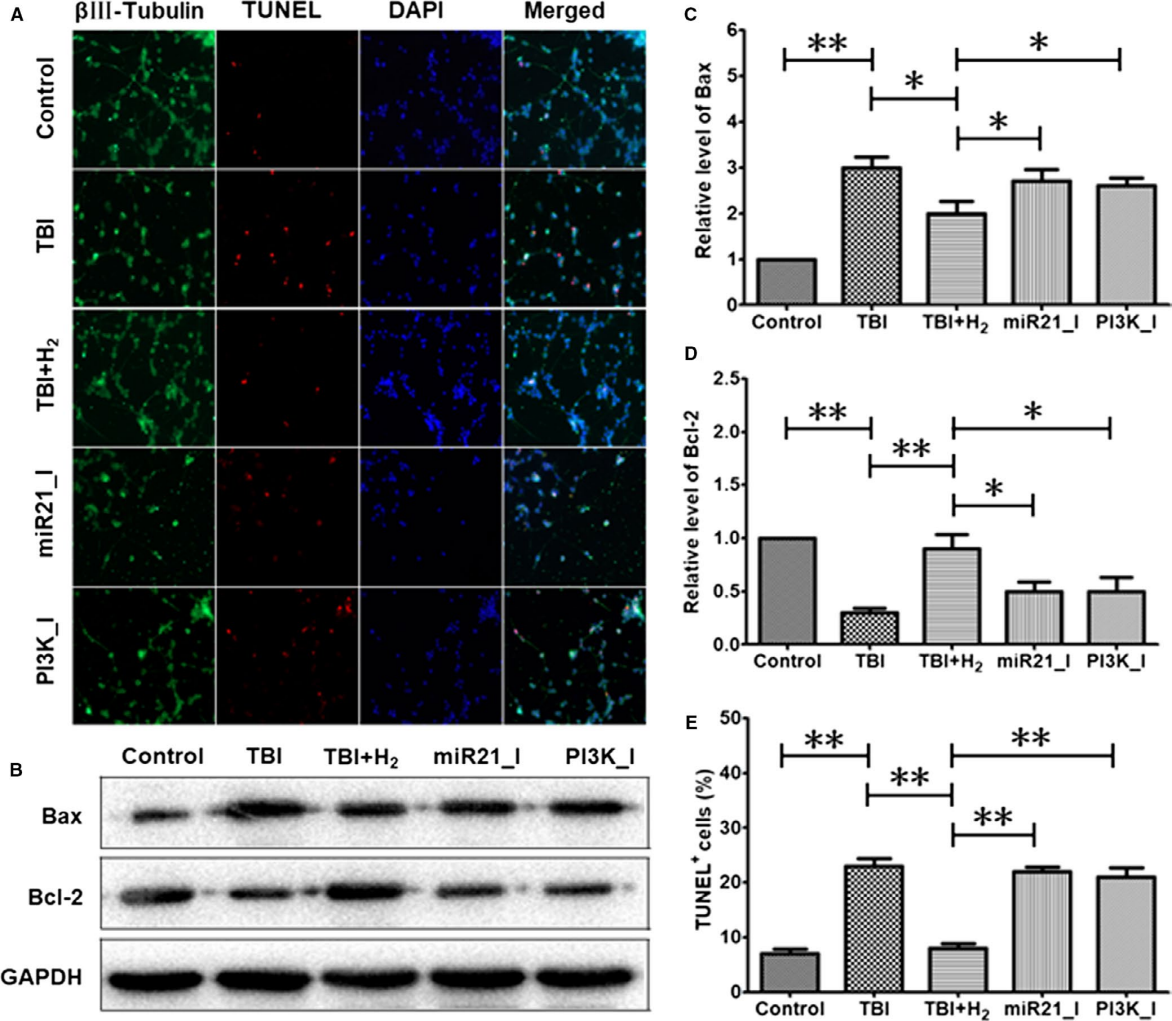
Expression levels of apoptosis‐related proteins and TUNEL staining in PC12 cells. Expression levels of Bax and Bcl‐2 proteins were measured using Western blot analysis in control cells or injured cells 48 h after scratching. Double immunofluorescence staining for βIII‐tubulin and TUNEL staining were adopted in control cells or injured cells at the same time point. The results were expressed as mean ± standard deviation. Differences in these markers among the five groups were compared using one‐way ANOVA with LSD *t* test. ‘*’ indicates *P* < .05, and ‘**’ indicates *P* < .01

Apoptosis was measured using TUNEL staining (Figure 6A,E). Apoptosis was increased in the TBI group compared with that in the control group (*P* < .05). After hydrogen intervention, apoptosis was significantly decreased in the TBI + H_2_ group compared with that in the TBI group (*P* < .05). In the miR21_I and PI3K_I groups, the miR‐21 antagomir and the PI3K blocker led to significant rises in apoptosis (*P* < .05 and *P* < .05, respectively).

As shown in Figure 6B‐D, the level of apoptosis protein Bax was higher in the TBI group than in the control group (*P* < .05). In the TBI + H_2_ group, the level of Bax was decreased compared with the TBI group (*P* < .05). However, in the miR21_I and PI3K_I groups, the level of Bax was elevated again (*P* < .05). In contrast, the level of anti‐apoptosis protein Bcl‐2 was lower in the TBI group than in the control group (*P* < .05). In the TBI + H_2_ group, the level of Bcl‐2 was increased compared with that in the TBI group (*P* < .05). However, in the miR21_I and PI3K_I groups, the expression of Bcl‐2 was decreased again (*P* < .05). These findings confirmed that hydrogen inhibited apoptosis partly through activation of miR‐21/PI3K/AKT/GSK‐3β signalling in injured cells.

### 3.7 Hydrogen relieved oxidative stress in injured cells partly through activation of miR‐21/PI3K/AKT/GSK‐3β signalling

**Figure 7:**
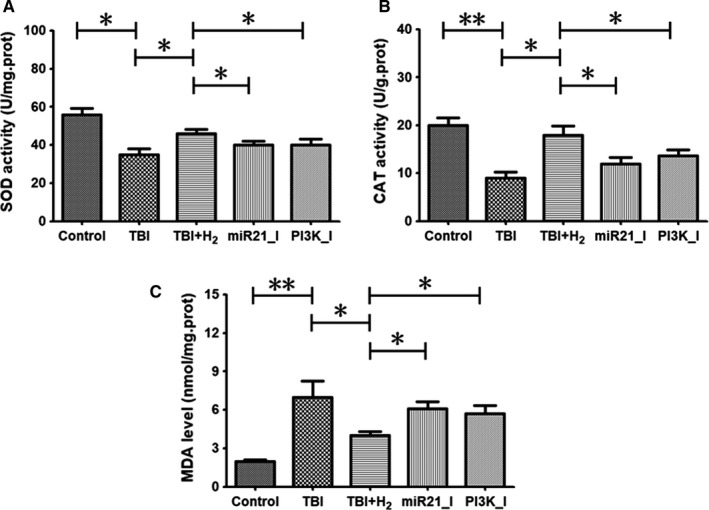
Oxidative stress levels in PC12 cells. Levels of superoxide dismutase (SOD), catalase (CAT) and malondialdehyde (MDA) in control cells or injured cells 48 h after scratching were measured using their commercial test kits. The results were expressed as mean ± standard deviation. Differences in these markers among the five groups were compared using one‐way ANOVA with LSD *t* test. ‘*’ indicates *P* < .05, and ‘**’ indicates *P* < .01

As illustrated in Figure 7A,B, the activities of antioxidant enzymes SOD and CAT were significantly decreased in the TBI group compared with those in the control group (*P* < .05 and *P* < .01, respectively). After hydrogen treatment, the activities of these two antioxidant enzymes were increased in the TBI + H_2_ group compared with the TBI group (*P* < .05 and *P* < .05, respectively). In the miR21_I and PI3K_I groups, the activities of SOD and CAT were decreased compared with the TBI + H_2_ group (miR21_I vs TBI + H_2_: *P* < .05 and *P* < .05, respectively; PI3K_I vs TBI + H_2_: *P* < .05 and *P* < .05, respectively).

Figure 7C shows that the level of oxidative stress product, MDA, was higher in the TBI group compared with that in the control group (*P* < .05). Compared with the TBI group, the level of MDA was significantly decreased in the TBI + H_2_ group (*P* < .05). In the miR21_I and PI3K_I groups, the level of MDA was higher than that in the TBI + H_2_ group (*P* < .05 and *P* < .05, respectively). These findings indicate that hydrogen inhibited oxidative stress partly through activation of the miR‐21/PI3K/AKT/GSK‐3β pathway.

## 4 DISCUSSION

This study revealed that hydrogen‐rich water elevated cell viability, relieved neuronal injury, improved neurite regeneration, inhibited apoptosis and promoted pro/anti‐oxidative stress balance in an in vitro model of TBI. The study also found that TBI was accompanied by the up‐regulation of miR‐21, p‐PI3K, p‐AKT and p‐GSK‐3β. Furthermore, a miR‐21 antagomir inhibited the expression of these four molecules, while a PI3K blocker only affected the expression of the three proteins and not miR‐21. More importantly, the miR‐21 antagomir and PI3K blocker both reversed the effect of hydrogen on neurons in this model. Taken together, hydrogen may exert its neuroprotective effect through activation of the miR‐21/PI3K/AKT/GSK‐3β signalling pathway in this in vitro model of TBI.

PC12 cells are used in many in vitro neurobiological studies because they retain the characteristics of dopaminergic neurons. Dopaminergic neurons are mainly distributed in the brain stem (midbrain) and have a wide range of important biological roles. Therefore, we purposefully adopted PC12 cells to explore the potential effect of hydrogen on brain stem trauma and injured dopaminergic neurons. Scratch modelling has been used in a large number of studies, including by ourselves.^18^, ^19^, ^20^, ^22^, ^23^ In the present study, we found significant abnormalities in cell viability, neurite regeneration, apoptosis and oxidative stress after scratching, which are typical molecular pathophysiological changes of TBI. Subsequently, we explored the effect of hydrogen on these changes and the potential mechanism involved.

Many studies have explored the potential effects of several medical gases on nerve regeneration, oxidative stress and apoptosis after TBI. Hyperbaric oxygen promotes nerve regeneration and attenuates apoptosis in animal models of TBI and patients.^24^, ^25^ Hydrogen sulphide offered neuroprotection after TBI in mice, together with reduced apoptosis and oxidative stress.^26^, ^27^ As another medical gas, hydrogen or hydrogen‐rich water has many important advantages compared with other medical gases. It is odourless, non‐toxic and easy to use. Hydrogen protects against oxidative stress and inflammation after TBI,^7^, ^8^ but few studies have focused on the effect of hydrogen on apoptosis and nerve regeneration after injury. In the present study, we observed decreased apoptosis and improved neurite regeneration after hydrogen treatment in an in vitro model of TBI.

miR‐21 is a non‐protein‐coding RNA molecule that is associated with the neurological outcome of TBI and that is affected by hydrogen treatment.^6^ However, the downstream effects of miR‐21 had not been elucidated. Feng et al^12^ suggested that miR‐21 attenuated amyloid‐β‐triggered apoptosis by modulating the PDCD4/PI3K/AKT/GSK‐3β pathway in SH‐SY5Y cells. Ahmad Rather et al^15^ reported that asiatic acid inhibited aluminium chloride–induced tau pathology, oxidative stress and apoptosis via the AKT/GSK‐3β signalling pathway in Wistar rats. Zhou et al^16^ revealed that epigallocatechin‐3‐gallate reduced apoptosis in substantia nigra neurons in Parkinson's model rats through mTOR/AKT/GSK‐3β activation. Therefore, miR‐21 has an ability to regulate PI3K/AKT/GSK‐3β signalling, and activation of AKT/GSK‐3β plays a protective role in many neurological diseases. Here, we confirmed that hydrogen exerted a series of neuroprotective effects by up‐regulating the expression of miR‐21 and further activating the PI3K/AKT/GSK‐3β pathway.

The cellular and molecular abnormalities caused by TBI were interrelated. Oxidative stress can directly damage neurons, resulting in neuronal injury and decreased cell viability. Oxidative stress can also induce apoptosis and indirectly damage neurons. All these pathological changes promote neuronal structural abnormalities and reduce neuron regeneration ability, resulting in deterioration of neurological function. Hydrogen plays a protective role in TBI partly through activation of the miR‐21/PI3K/AKT/GSK‐3β signalling pathway, which can be used as a theoretical basis for the relationships among these abnormalities.

GSK‐3β is an evolutionarily conserved serine/threonine kinase that can act on many signalling proteins and transcription factors to regulate cell differentiation, proliferation, survival and apoptosis. Therefore, GSK‐3β is considered a therapeutic target for a variety of nervous system diseases. Some studies using in vivo or in vitro models indicate that GSK‐3β may regulate neuronal apoptosis through activation of NF‐κB, β‐catenin or CREB signalling pathways.^28^, ^29^, ^30^ Other studies report that GSK‐3β can activate Nrf2 signalling to attenuate β‐amyloid‐induced oxidative stress in Alzheimer's disease models.^31^, ^32^ Therefore, the role of GSK‐3β and its downstream pathways in hydrogen treatment against TBI should be fully investigated in the future.

In conclusion, the present study revealed the protective effect of hydrogen‐rich medium on neurite regeneration, neuronal apoptosis and oxidative stress induced in an in vitro model of TBI. We also confirmed the existence of the miR‐21/PI3K/AKT/GSK‐3β signalling pathway in PC12 cells, and the neuroprotective role of hydrogen was mediated in part by activation of this pathway.

## CONFLICT OF INTEREST

The authors confirm that there are no conflicts of interest.

## AUTHOR CONTRIBUTIONS

Wang Lu, Yin Zhenyu, Wang Feng and Lei Ping designed the study. Wang Lu, Yin Zhenyu, Wang Feng, Wang Yifeng and Hu Tianpeng carried out literature research. Wang Lu, Yin Zhenyu, Wang Feng, Huang Shan, Hu Tianpeng, Guo Mengtian and Han Zhaoli carried out experiment. Wang Yifeng, Huang Shan, Hu Tianpeng and Guo Mengtian analysed and interpreted the data. Wang Yifeng, Huang Shan and Han Zhaoli performed statistical analysis. Wang Lu, Yin Zhenyu, Wang Feng, Guo Mengtian and Han Zhaoli prepared the manuscript. Wang Lu, Yin Zhenyu, Wang Feng and Lei Ping approved the final version of the manuscript. Wang Lu and Han Zhaoli revised the manuscript.

## Data Availability

Data cannot be shared because this is an ongoing study.
